# The role of *lrp-2* in *C. elegans* vulval cell lineage polarity

**DOI:** 10.17912/micropub.biology.000155

**Published:** 2019-08-27

**Authors:** Paul J Minor, Paul W Sternberg

**Affiliations:** 1 Division of Biology and Biological Engineering, Caltech, Pasadena, CA 91125 pws@caltech.edu; 2 Department of Biology, Hopkins Marine Station of Stanford University, Pacific Grove, CA 93950. paul.j.minor@gmail.com

**Figure 1. Model for the role of LRP-2 in vulval lineage cell polarity:**
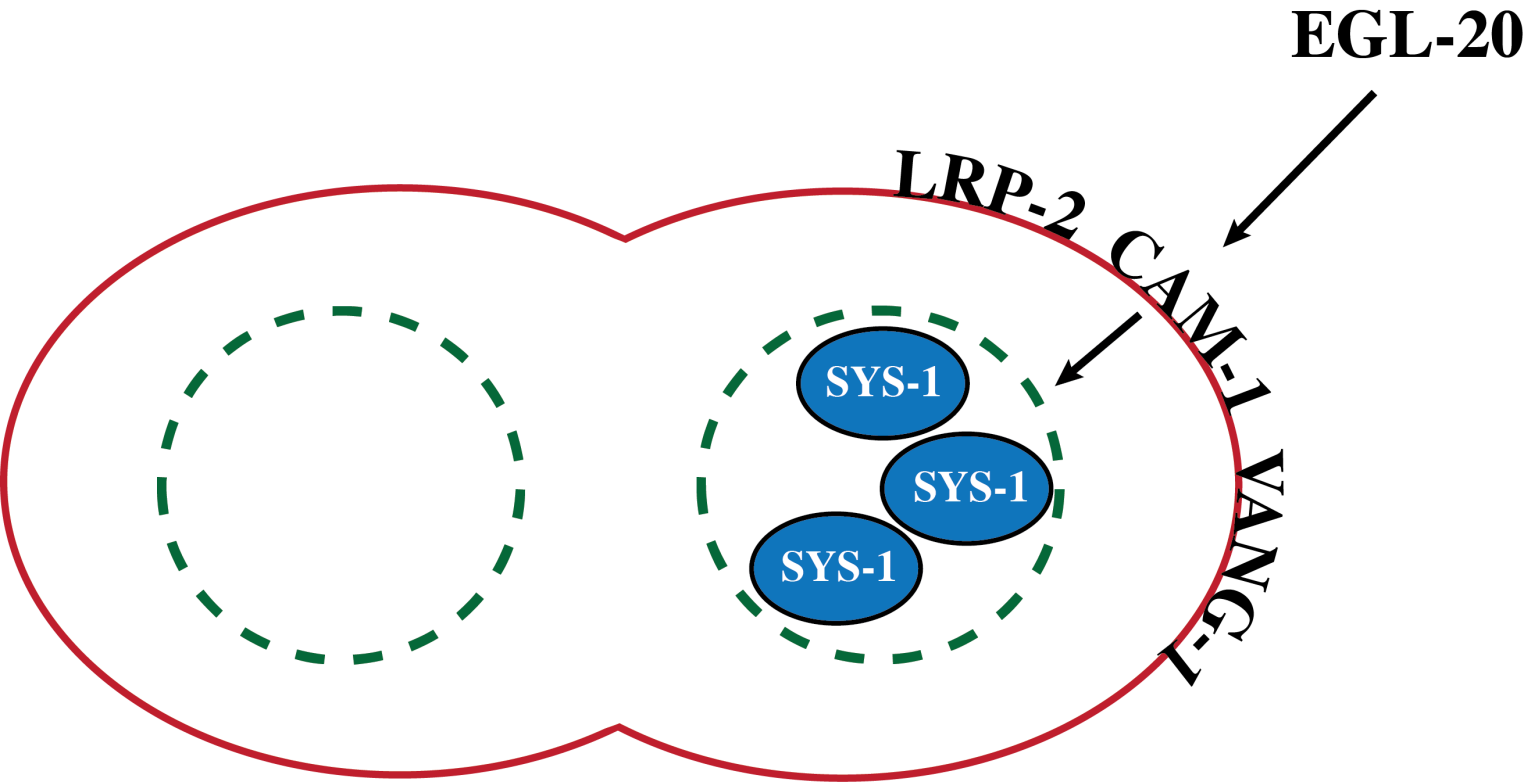
LRP-2 is proposed to work with CAM-1 and VANG-1 to respond to the Wnt protein EGL-20 and stabilize SYS-1 in the posterior daughter of a vulval precursor cell.

## Description

We investigated the role of the low-density lipoprotein receptor *lrp-2* in *C. elegans* vulval cell lineage polarity. We find that despite the high conservation of the Wnt signaling component, LRP5/6, in higher order organisms it appears to have evolved after the split of Nematoda due to its absence in all nematode genomes examined. *C. elegans* contains multiple low-density lipoprotein receptors within its genome, two of which are *lrp-1* and *lrp-2*. Due to the position in the genome and high sequence similarity we believe that *lrp-2* is the product of a recent duplication of *lrp-1* within the *Caenorhabditis* lineage (Minor and Sternberg, 2019d).

To understand the potential role of *lrp-1* and *lrp-*2, we sought to score the vulval phenotypes of these mutants during L4 stage. *lrp-1(ku156)* mutants are sick and arrest during an early larval stage. For this reason we were not able to examine the role of *lrp-1* in vulval formation. Despite high sequence similarity and proposed functional redundancy with *lrp-1*, *lrp-2* mutants are remarkably wild-type. *lrp-2* is expressed in the developing vulva at the same time as both *cam-1* and *vang-1*. By examining double and triple mutant strains we find that *lrp-2* is likely to function downstream of *egl-20* along with transmembrane proteins *cam-1* and *vang-1* (Minor and Sternberg, 2019b; Minor and Sternberg, 2019c). All three genes antagonize the *lin-17*/Frizzled pathway by directing the aberrant localization of SYS-1 to the posterior daughter cell of P7.p, thereby leading to the posterior orientation of the P7.p lineage (Minor and Sternberg, 2019a). These observations suggest the simple model shown in Figure 1, in which LRP-2 cooperates with CAM-1 and VANG-1 to respond to EGL-20.

This work provides evidence that despite lacking a true LRP5/6 ortholog, the formation of the *C. elegans* vulva is controlled by another member of the low-density lipoprotein superfamily, *lrp-2*. This data could potentially lead to insight into the evolution of both structure and function of the highly important Wnt pathway component, LRP5/6. Despite strong genetic evidence, this work does not describe the physical interaction between LRP-2 and CAM-1, VANG-1, EGL-20. Can LRP-2 bind with the other transmembrane proteins, CAM-1 and VANG-1, involved in this pathway? Can LRP-2 physically bind the Wnt ligand, EGL-20? Future work should focus on the biochemistry of this pathway. Answers to these questions could provide interesting insights into the evolution of low-density lipoprotein receptors, including LRP5/6, as well as how Wnt signaling has evolved within nematodes without the presence of one of the most important and highly conserved transmembrane proteins.

## Reagents

**Strains:** Strains obtained from the CGC and provided by the *C. elegans* Reverse Genetics Core Facility at the University of British Columbia, which is part of the international *C. elegans* Gene Knockout Consortium.

**MH210**: *lrp-1(ku156)/gld-1(q266)*

**VC621**: *lrp-2(gk292)*

**VC543**: *lrp-2(gk272)*

## References

[R1] Minor Paul J, Sternberg Paul W (2019). LRP-2 controls the localization of *C. elegans* SYS-1/beta-catenin. microPublication Biology.

[R2] Minor Paul J, Sternberg Paul W (2019). *C. elegans* LRP-2 functions in vulval precursor cell polarity. microPublication Biology.

[R3] Minor Paul J, Sternberg Paul W (2019). LRP-2 likely acts downstream of EGL-20/Wnt. microPublication Biology.

[R4] Minor Paul J, Sternberg Paul W (2019). Low density lipoprotein receptors LRP-1 and LRP-2 in C. elegans. microPublication Biology.

